# Differences between Monolinguals and Bilinguals in Phonetic and Phonological Learning and the Connection with Auditory Sensory Memory

**DOI:** 10.3390/brainsci13030488

**Published:** 2023-03-14

**Authors:** Laura Spinu, Jiwon Hwang, Mariana Vasilita

**Affiliations:** 1 CUNY-Kingsborough Community College, Brooklyn, NY 11235, USA; 2 CUNY-The Graduate Center, New York, NY 10016, USA; 3 Stony Brook University, Stony Brook, NY 11794, USA

**Keywords:** bilingualism, auditory sensory memory, phonetic and phonological learning

## Abstract

Bilingualism has been linked with improved function regarding certain aspects of linguistic processing, e.g., novel word acquisition and learning unfamiliar sound patterns. Two non mutually-exclusive approaches might explain these results. One is related to executive function, speculating that more effective learning is achieved through actively choosing relevant information while inhibiting potentially interfering information. While still controversial, executive function enhancements attributed to bilingual experience have been reported for decades. The other approach, understudied to date, emphasizes the role of sensory mechanisms, specifically auditory sensory memory. Bilinguals outperformed monolinguals in tasks involving auditory processing and episodic memory recall, but the questions whether (1) bilinguals’ auditory sensory memory skills are also enhanced, and (2) phonetic skill and auditory sensory memory are correlated, remain open, however. Our study is innovative in investigating phonetic learning skills and auditory sensory memory in the same speakers from two groups: monolinguals and early bilinguals. The participants were trained and tested on an artificial accent of English and their auditory sensory memory was assessed based on a digit span task. The results demonstrated that, compared to monolinguals, bilinguals exhibit enhanced auditory sensory memory and phonetic and phonological learning skill, and a correlation exists between them.

## 1. Introduction

For decades, the psycholinguistic literature has reported the existence of a bilingual cognitive advantage [1,2] whereby bilingual language experience is thought to enhance cognitive functions and ultimately contribute to cognitive reserve. However, the bilingual advantage has polarized academics as a controversial, difficult to replicate phenomenon, earning the nickname of “Loch Ness monster” [3,4]. In a different meta-analysis based on 46 original research studies, Van den Noort et al. [5] report that a majority of the articles on the topic (54.3%) found beneficial effects of bilingualism on cognitive control tasks; however, 28.3% found mixed results and 17.4% found evidence against its existence. Following DeLuca et al. [6], we take the position that bilingual effects on cognition exist, but they are conditional. It is no coincidence that the 2021 meeting of the world’s largest conference on bilingualism, the International Symposium on Bilingualism, hosted two theme sessions entitled *Biases in research: Who counts as ‘authentic’ bilingual speaker—and how can we tell?* and *Language proficiency measures—what exactly are we measuring?* Several reasons, both methodological and conceptual in nature, have been invoked as potentially underlying the conflicting bilingual advantage findings [7,8]. These include individual differences such as talent [9], language-pair factors [10], the fact that the bilingual advantage may be most prominent during early and late stages of life, but less noticeable during adulthood [11], and experimental task complexity across studies [2]. Among these factors, the fact that all speakers have access to non-linguistic ways of improving cognitive function, the lack of a well-defined operational description of bilingualism, and the omission of lower-level, sensorimotor functions in considering the relationship between language and cognition have received heightened attention in recent literature. It is the third aspect we address in more detail in the current paper.

As mentioned above, numerous studies on bilingual cognition have explored the potential advantages associated with bilingualism on executive function. As Poarch and Krott [12] explain, the view that bilingualism has cognitive benefits is based on the theoretical assumption that bilingual individuals experience constant cross-linguistic activation and interaction during language processing [13,14]. To enable the use of the correct language in a given context, the need arises for a cognitive control mechanism permitting speakers to resolve the conflict between languages that are actively competing with each other. Such a cognitive control mechanism already exists for non-verbal processing, specifically executive function(s) [7,15]—also referred to generically as cognitive control. Executive function refers to a set of processes considered necessary for the cognitive control of behavior, including (in most models) attentional control, inhibitory control, working memory, and cognitive flexibility or shifting. Because frequent switching between languages is speculated to employ this mechanism, the expectation arises that the more this happens, the greater the enhancement of cognitive function [16]. Miyake et al. (2000) investigated the separability of shifting, updating, and inhibition, reporting that these three executive functions have differential contributions to performance on complex frontal lobe tasks [17]. Given that the frontal lobes are involved in language processing [18,19] and brain adaptations have been observed in the frontal regions in bilinguals [20], executive functions are likely to be involved in multiple aspects of language learning [6], though other mechanisms are likely to be involved as well.

**Figure 1:**
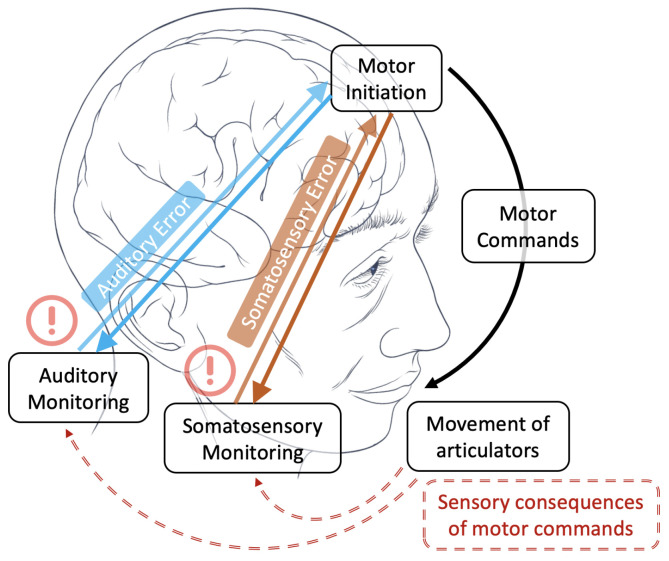
Sensorimotor systems involved in speech (adapted here from [21]).

Over a decade ago, Simmonds et al. posed the question why previous bilingualism research had largely ignored sensorimotor aspects of learning [21]. Indeed, an understudied area of research pertaining to bi- and multilingualism is their connection with cognitive aspects outside of the frequently explored set of executive functions. Because, as shown in Figure 1, language experience involves extensive use of sensorimotor mechanisms [21,22], such as motor (articulatory) control, somatic memory, and auditory sensory memory (iconic memory in the case of signed languages), the question arises whether these lower-level functions are also enhanced by bilingual experience outside of one’s native language. Furthermore, if that is the case, the contribution of sensorimotor functions to cognitive function and whether a connection exists between sensorimotor and executive functions also needs to be clarified. Lindenberger (1994) and Lindenberger et al. (2000) posited a connection between the two in the cognitive permeation hypothesis [23,24], noting that sensorimotor aspects of behavior are more attention-demanding in older adults than in young adults, which leads to increased competition between sensorimotor and cognitive tasks for scarce attentional resources. Reviewing the research on the coupling between sensorimotor and cognitive aging, Schäfer et al. (2006) conclude that they are causally related and functionally interdependent and that age-associated increments in cognitive resource demands of sensorimotor functioning are malleable by experience [25]. Their recommendation is for future studies to attempt to shed further light on functional and etiological links between sensorimotor and cognitive aging and their interaction.

Exploring the connection between sensorimotor and cognitive functions also has the potential to shed more light on a phenomenon that has received heightened attention recently, specifically phonetic and phonological learning. Experimental research has shown that bilingual individuals (of various backgrounds) tend to outperform monolinguals in tasks requiring them to produce or perceive novel sounds or accents of a known language. For instance, ref. [2] trained monolinguals and bilinguals on vocabularies differentiating words that contained foreign phonetic contrasts. Their findings suggested a bilingual advantage in phonetic learning, which is influenced by the level of difficulty of the specific phonetic contrast being learned and by the similarity between the learners’ native language and the target language (a similar conclusion was drawn by [26] in their study that investigated the acquisition of rhotics longitudinally). In a study focusing on non-native contrasts, ref. [27] report enhanced speech perception abilities in multilinguals and bilinguals compared to monolinguals, whose ability to discriminate a non-native contrast did not differ from that of the bilingual and multilingual group before training).

Using a more naturalistic approach focusing on the global learning of a different accent and thus expanding on the production or perceptual discrimination studies employing sounds in isolation, ref. [28] compared Canadian monolinguals and bilinguals in an experiment that involved two tasks: imitating and spontaneously reproducing a novel foreign accent spoken in Sussex, England. The target sound (i.e., the glottal stop), which was already present in the speakers’ production, was mapped differently to surface forms in the novel accent (i.e., as the only allophonic realization of word-final coronal stops). The results suggest more effective learning in bilinguals. Although the two groups performed very similarly during the training when they were asked to imitate what they heard immediately, bilinguals produced their glottal stop significantly more frequently than monolinguals during the post-training session. A follow-up study [29] employed a novel accent that was artificially created to have four phonetic features that differed from standard American English. The decision to use an artificially constructed accent instead of a natural one was made to allow better control over the measurements of the input and the output in the experiment. Early bilinguals of various language backgrounds consistently outperformed monolinguals. These findings are in line with a bilingual advantage found in phonetic and phonological learning that is robust enough to override the various issues speculated to cause conflicting results in the executive function studies discussed previously. Departing from the more widespread executive function work, we address the question whether a link exists between phonetic and phonological learning and auditory sensory memory. Given the complexity of phonetic and phonological learning, we expect it to be underlain by multiple mechanisms, including executive function, but in the current paper we narrow down the investigation to auditory sensory memory precisely because this connection has been understudied to date.

Turning to the work on auditory sensory memory, the digit span task (with a suffix) is a paradigm commonly employed to investigate this type of memory in behavioral studies. The suffix effect, as described by previous studies [30,31], refers to the difficulty in recalling a spoken sequence caused by the addition of an irrelevant speech item at the end. Typically, participants are presented with sequences of digits or letters that are arranged in a random order, followed by either a silent interval [32] or a suffix of equivalent duration (e.g., the word “go”). When compared to the items followed by a silence, the items closest to the suffix display an increase in errors, with the final item showing the largest increase in errors. This is in contrast with near-perfect performance in the control condition. Replicated consistently in a variety of studies, the suffix effect is thought to reflect an automatic type of processing that is characteristic of the functioning of auditory sensory memory [33,34,35].

It should be added that other types of memory, such as working memory, are likely active in digit span recall [36]. It is believed that information about the stimulus heard most recently can be accessed simultaneously by both auditory sensory memory and working memory. As a result, it can be challenging to differentiate the effects of auditory sensory memory and those of working memory processes, such as rehearsal, long-term retrieval, or chunking [37]. However, empirical studies have been able to distinguish the separate effects of working memory rehearsal and auditory sensory memory to digit span recall, along with their accompanying theoretical interpretations [38,39]. The general view has been that auditory serial recall tasks enable the separation of performance effects resulting from working memory rehearsal, which affects the first items in a longer list (i.e., primacy effects), from performance effects resulting from auditory recency, which applies to the last items in a list (i.e., recency effects). Based on this, we consider performance on the terminal items of a list mainly to reflect the working of auditory sensory memory, while not excluding the possibility of interference from additional mechanisms interacting with it, such as working memory where they need to hold incoming L2 information while decoding it. One should note, however, that recent work by Sofologi et al. [40] showed no differences in working memory between monolingual and bilingual students of the same age, while at the same time finding a bilingual advantage in inhibitory control and cognitive change. The authors conclude that when learning a (first or second) language, working memory does not correlate to all executive functions but forms a separate cognitive function. These findings are supported by Yang’s 2017 study [41], which concluded that knowing two languages does not guarantee bilingual working memory advantages over monolinguals, but the advantage might be linked to bilinguals’ unique L2 use environment. On the other hand, the relationship remains unclear: Morales et al. [42] found an advantage for bilingual children in working memory that was especially evident when the task contained additional executive function demands.

While research has shown a bilingual advantage in tasks involving auditory processing [43] and episodic memory recall [44], very few studies have investigated auditory sensory memory in the context of bilingualism. Philipp-Muller et al. [45] administered a digit recall task and used an algorithm to analyze the digit recall data and examine the mechanism underpinning the differences in memory performance in bilingual and monolingual participants. The Rational Transpositional Error Algorithm (RTEAlgorithm) showed that bilinguals made significantly fewer transpositional errors than monolinguals in the recall task. This study, however, did not specifically investigate performance on the terminal items of digit sequences and therefore its findings are not conclusive with respect to auditory sensory memory. More recently, ref. [46] administered a suffixed adaptive digit span task to bilinguals and monolinguals from the undergraduate population of the University of Toronto, and compared them in overall accuracy, accuracy by serial position, maximum number of digits recalled, and the percentage of participants who reached the longest digit span. The results showed that bilinguals have longer digit spans and higher accuracy than monolinguals across all serial positions within every list length. This suggests an advantage for bilinguals not only in terms of recently heard items, which are attributable to auditory sensory mechanisms (known as recency effect), but also for the items heard at the beginning of longer list lengths, which are owed to working memory (known as primacy effect). While [46] concluded that bilingual experience results in enhanced auditory sensory memory, further studies are needed to consolidate this finding and to explore the connection between this type of memory and phonetic and phonological learning, especially as the former has been suggested to have a significant role in the latter [18].

In sum, based on the research on phonetic and phonological learning and auditory sensory memory, which were both found to be enhanced in bilinguals, it is plausible to assume a link between the two, and further speculate that the mechanism supporting phonetic and phonological learning is partially supported by the work of auditory sensory memory. The experiment described in the following sections addresses the possible existence of a correlation between phonetic and phonological learning and auditory sensory memory.

## 2. Experiment: Materials and Methods

The aim of the current study was to address the prediction put forth in the previous section, which postulates a link between phonetic and phonological learning and auditory sensory memory, an experiment was designed to include a novel accent learning task, following [28,29] and a digit span task with a suffix [46]. The experiment was conducted with monolingual and bilingual speakers in person in a quiet room, inside a sound-attenuated booth, on the CUNY Kingsborough Community College campus and comprised the following parts: a language background questionnaire, a translation task for bilingual participants (from English into their other language)—not discussed here, a novel accent learning task that included three blocks (i.e., baseline, training, and testing), and a digit span task with a suffix. Preliminary findings of this study (covering a subset of the participants and only the results obtained for the phonetic and phonological learning task) were reported in [29].

### 2.1. Hypotheses

Our predictions are primarily based on previous findings suggesting that there is an advantage for bilinguals in phonetic and phonological learning [2,27,28,29] and in serial memory tasks [45], including those specifically focusing on auditory sensory memory [46].

**Hypothesis** **1.** *Bilinguals will outperform monolinguals on the phonetic and phonological learning tasks.*

**Hypothesis** **2.** *Bilinguals will display enhanced auditory sensory memory compared to monolinguals.*

**Hypothesis** **3.** *A significant correlation exists between auditory sensory memory and phonetic and phonological learning.*

### 2.2. Language Background Questionnaire

Participants were individually administered an abbreviated version of the LEAP-Q questionnaire [47]. Following [46], participants who were included in the bilingual group met two primary criteria: (1) self-reported native or near-native proficiency level in both languages, and (2) exposure to both languages prior to school age (i.e., 6–7 years). Monolinguals were defined as individuals who reported speaking English natively and, in some cases, a second language at a level of conversational or beginner proficiency.

### 2.3. Testing Phonetic and Phonological Learning: The Novel Accent Learning Task

#### 2.3.1. Stimuli

**Figure 2:**
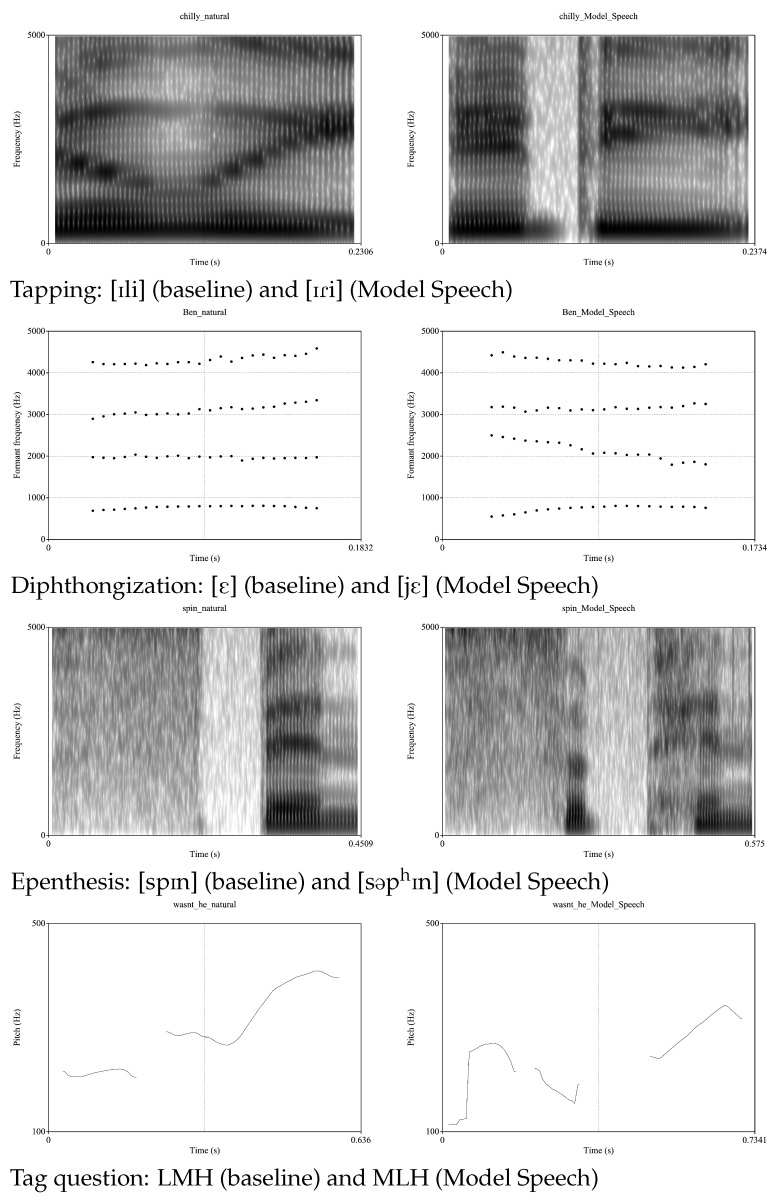
Examples of the 4 features in baseline (**left**) and Model Speech (**right**). The VCV sequence for tapping was extracted from the word ‘chilly’. The tracks of formants 1–4 are obtained from the vowel in the word ‘Ben’. The spectrograms for epenthesis are obtained from the word ‘spinning’. Pitch tracks for the sequence ‘wasn’t he?’ illustrate the intonation change feature.

An artificial accent of English (henceforth Model Speech), was created such that it differed in four distinct ways from standard North American English (Figure 2):

**1.**  **Tapping:** intervocalic /l/ → [ɾ] e.g., ‘color’ →[kʌɾɚ]
**2.**  **Diphthongization:** the vowel /ɛ/ → [jɛ] after an onset consonant, e.g., ‘bed’ → [bjɛd]
**3.**  **Vowel epenthesis:** voiceless clusters of the form sC̥ → səC̥ e.g., ‘spy’ → [səp^h^aj]
**4.**  **Intonation change:** tag questions were realized with a novel Mid-Low-High (MLH) pattern. Tag questions (e.g., *isn’t it?*) are typically produced with either rising or falling intonation in standard American English.

The stimuli consisted of short sentences containing either one single feature e.g., *You make a good spy*, where *spy* was realized as [səp^h^aj] (epenthesis), two features combined e.g., *She put a spell on him*, where [spɛl] was realized as [səp^h^jɛl] (epenthesis and diphthongization), or all four of them (e.g., *You set the speed alone, didn’t you?* where the vowel in the word *set* was diphthongized, epenthesis occured in the word *speed*, tapping affected the [l] in *alone*, and the tag question *didn’t you?* was realized with a MLH contour).

The features were distributed as follows: 20 tapped /l/, 20 diphthongized vowels, 20 epenthesized vowels and 10 tag questions. The reason we included a lower number of tag questions compared to the other novel features was that they were found impressionistically to be highly salient and their presence in higher numbers was deemed to have a distracting effect on the listeners.

The total list of stimuli comprised 40 sentences (of which 20 contained single features, 15 contained combinations of two features, and 5 contained all four features). A highly trained monolingual female phonetician recorded the full list of stimuli using the Model Speech and also in her natural Northeastern US accent (for comparison). The consistent presence of all novel features in the artificial accent was verified acoustically (see Figure 2).

#### 2.3.2. Procedure

The experimental procedure for this task started with the recording of 40 baseline sentences containing all structures of interest, followed by a two-part training phase. In the first part, participants listened to 40 sentences spoken in the Model Accent continuously, in the absence of orthographic input. In the second part, they listened to each of the same 40 sentences and were asked to immediately imitate it in the novel accent (see [48] for the role of imitation in phonetic and phonological learning), while also being able to see its orthographic transcription on a computer screen. In the testing phase, they read the baseline sentences again, this time aiming to reproduce the novel accent without any audio prompts. This task was administered using PsychoPy [49].

#### 2.3.3. Data Processing and Analysis

Data processing consisted of categorical judgments provided by the same trained monolingual phonetician who recorded the Model Speech sentences (Note: the rater is not an author and had obtained her PhD prior to her collaboration on this study). The absence or presence of each target feature was scored with a 0 or 1, respectively, resulting in a mean accent score for each participant and for each block, as well as an overall score per participant averaging over the three blocks (baseline, training/imitation, and testing). While the scoring process was not blind, with the rater having access to language background information for the participants, the judgments were based on spectrographic evidence (as shown in Figure 2) and not on impressionistic data. While we anticipate conducting a number of acoustic analyses to be reported in a future study, including measurements of continuous parameters such as duration, pitch and formant values, as well as other pertinent measures for each of the four features employed, the current study is based on the categorical ratings only. The statistical analyses we conducted for the current study include a series of ANOVAs that compared various aspects of the two groups’ performance across the different features, blocks, and sentence types (that is, containing 1, 2, or 4 features together), detailed in the following sections. See Appendix A for a detailed description of the variables employed.

### 2.4. Testing Auditory Sensory Memory: The Digit Span Task with Suffix

#### 2.4.1. Stimuli

The stimuli consisted of sequences of digits varying in length (from a minimum of 2 digits to a maximum of 9). After each digit sequence, the word “recall” was presented, which served as a suffix. Both the digits (1 through 9) and the suffix (i.e., “recall”) were generated using a natural-sounding synthetic male voice. The task was adaptive, presenting digit sequences of a specific length in blocks of five trials each. For example, a listener was first presented with 5 trials of 2-digit sequences, then 5 trials of 3-digit sequences, and so on and so forth, until they were no longer able to correctly recall at least 3 out of the 5 trials within a block. At that point, the task was terminated. Thus, the task could end earlier for some listeners compared to others, depending on their performance.

#### 2.4.2. Procedure

The default template of PsyScope [50] digit span was modified to construct this task. The task was designed to be adaptive, beginning with a practice block of two digits and progressing to longer sequences if the participant accurately recalled at least three of the five trials at each sequence length. As a result of the adaptive nature of the task, the highest sequence length achieved varied among participants, resulting in a different number of blocks presented depending on their individual memory capacity.

#### 2.4.3. Data Processing and Analysis

The software (PsyScope) automatically generated scores for each sequence, including the number of correct and incorrect responses and the maximum digit sequence length reached by each participant. Overall accuracy for each serial position for the longer digit sequences was subsequently obtained. A MATLAB script [51], specifically developed to compare the digits presented at each serial position with the participants’ response and determine accuracy based on whether a match was found was also used. The algorithm searched for insertions or deletions by aligning a participant response string and the input string presented and counting the number of digits in each string to see if there was a discrepancy. If the number of digits in the response string was equal to the number of digits in the input string, then the answer was included in the analysis, but if the number of digits was not equal between the response and input strings, the answer was excluded. The algorithm evaluated the responses that were included digit-by-digit, employing a graded scoring method that assigned weighted scores based on transpositional distance. The goal of this graded scoring system was to award a higher score to transposed response digits that were closer to their original position in the participant response.

The z-scores were used to compare the proportion of participants from each group who were able to reach the longest digit sequence (i.e., nine digits). In a series of ANOVAs, group (monolingual/bilingual) and sequence length (2 through 9) were included as the independent factors and digit span (i.e., a single score per subject consisting of the highest list length reached), accuracy, and the algorithm score as the dependent variables.

Lastly, correlation analyses were performed to identify any potential relationships between the accent scores obtained (both on separate blocks—training and testing—and overall) and digit accuracy, maximum digit length reached, and both the raw and algorithm-based scores. All variables of interest are described in Appendix A.

### 2.5. Participants

The participants were 62 undergraduate students, 31 monolingual (mean age = 23.6, SD = 6.08, 8 male, 23 female) and 31 early bilingual (mean age = 22.33, SD = 4.6, 9 male, 22 female). As previously described in Section 2.2, early bilingual participants were characterized by a native or near-native level of proficiency in both languages and early exposure to them, defined as prior to school age (i.e., 6–7 years). Bilinguals’ other languages included Arabic, Cantonese, Hebrew, Russian, Spanish, Urdu, Thai, and (Haitian/Jamaican/St. Lucian) Creole. Both age of acquisition and proficiency level were self-reported. Monolinguals were defined as individuals who reported speaking English natively and, in some cases, an additional language at a conversational or beginner level. Two of the participants (one from each group) were excluded from the analyses related to phonetic and phonological learning (and consequently the correlation analyses) due to technical issues leading to the loss of their voice recordings for the novel accent learning task, but their data were included in the analyses associated with auditory sensory memory.

### 2.6. Results

The results of the study are presented in three separate subsections, the first two reporting the findings for each of the two experimental tasks, and the third presenting the correlations between phonetic and phonological learning and auditory sensory memory.

#### 2.6.1. Phonetic and Phonological Learning: The Novel Accent Learning Task

**Figure 3:**
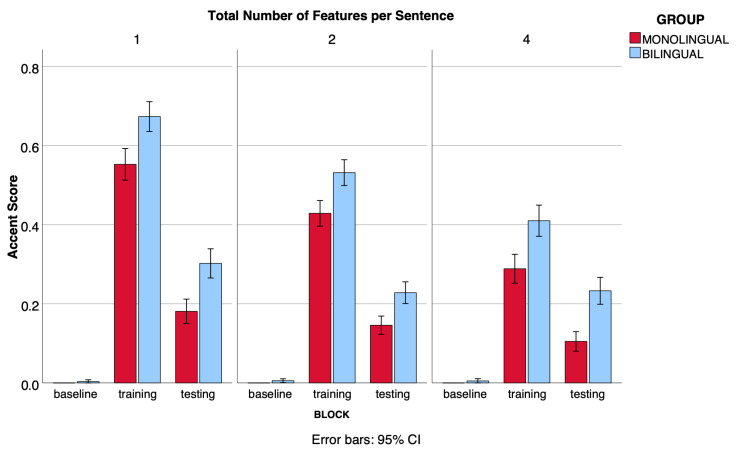
Mean of accent scores grouped by the number of novel accent features (1, 2, or 4) per sentence obtained by monolinguals and bilinguals in Baseline, Training and Testing.

Figure 3 shows the average scores for monolinguals and bilinguals grouped by the number of novel accent features (1, 2, or 4) in the three different conditions (i.e., Baseline, Training, Testing). Bilinguals outperformed monolinguals across the board, in both the Training (imitation) and Testing conditions, with a more pronounced decrease in performance for monolinguals in Testing as the number of features present per sentence increased.

**Figure 4:**
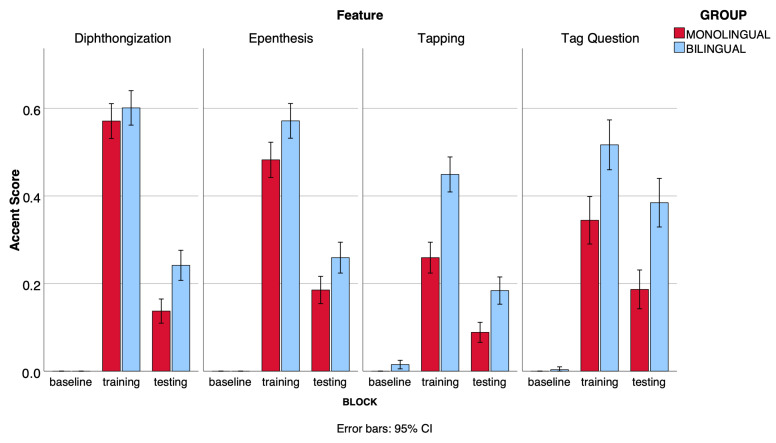
Mean of accent scores for each novel accent feature obtained by monolinguals and bilinguals in Baseline, Training and Testing.

Figure 4 shows the average scores for monolinguals and bilinguals for all four novel features in the three different conditions (i.e., Baseline, Training, Testing). Bilinguals outperformed monolinguals across the board, in both the Training (imitation) condition (except for the diphthongization feature) and the Testing condition, but the differences in Training were more pronounced with tapping and tag questions. In Testing, monolinguals performed best with tag questions, followed by epenthesis, and performed most poorly on the tapping feature. An ANOVA with *Accent Score* as the dependent variable and *Group (monolingual/bilingual)*, *Block (baseline/training/testing)*, *Feature (dipthongization/tapping/epenthesis/tag question)* and *Number of features per sentence (1/2/4)* as independent variables revealed significant main effects of all independent variables (Group: F(1, 12587) = 148.98, *p* < 0.001, Block: F(2, 12587) = 1768.32, *p*< 0.001, Feature: F(3, 12587) = 50.12, *p*< 0.001, and Number of features per sentence F(2, 12587) = 89.81, *p*< 0.001), and also of the interactions between Group × Block, Group × Feature, Block × Feature, Block × Number of features per sentence, Feature × Number of features per sentence, Group × Block × Feature and Block × Feature × Number of features per sentence. Post hoc tests using the Bonferroni correction revealed that each block differed significantly from the other two, and tapping differed significantly from all other features. The three configurations for number of features per sentence (1, 2, or 4) also differed significantly from each other.

#### 2.6.2. Auditory Sensory Memory: The Digit Span Task with Suffix

**Figure 5:**
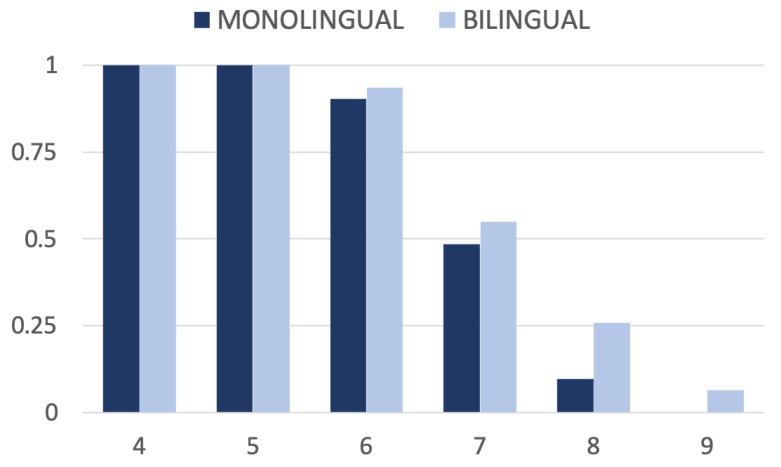
Digit span task: proportion of group who reached each list length.

Figure 5 presents the proportion of participants (monolingual or bilingual) who were able to advance to each sequence length. Participants from both groups began to “drop out” at sequence length = 6, but those from the monolingual group dropped out in greater proportions than the bilinguals—a slight difference at first, with 93.5% of bilinguals and 90.3% of monolinguals reaching sequence length = 6, which becomes larger as the sequence length increases, with 54.8% of bilinguals and 48.4% of monolinguals reaching sequence length = 7. Only 25.8% of bilinguals and 9.7% of monolinguals were able to complete successfully the 7-digit block and move to the 8-digit block. Only participants from the bilingual group moved on to the 9-digit block. However, none of these consistently recalled these sequences, which means that the 8-digit sequence was the longest sequence recalled reliably by participants in this experiment. Based on the use of a z-score to evaluate the proportions of the two populations still present at the 8-digit sequence (z = 1.6622, one-tailed), we conclude that significantly more bilinguals reached this list length compared to monolinguals (*p* < 0.05).

**Figure 6:**
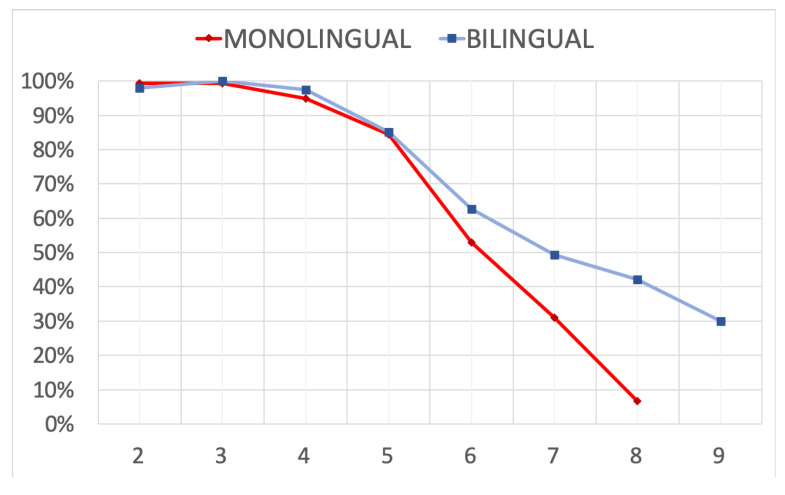
Memory task: mean accuracy for monolinguals and bilinguals for each sequence length.

The two groups did not differ significantly with respect to the average maximum digit length reached, which was 6.8 for bilinguals and 6.5 for monolinguals. For a finer-grained perspective, Figure 6 displays the two groups’ accuracy broken down by sequence length. As previously described, there were five trials in each block for a given list length, and a participant needed to answer at least 3 (out of the 5 trials) correctly in order to advance to the next (higher) sequence length. This means that even when a sequence length has been successfully completed by all of the participants, overall accuracy for that sequence length is not necessarily 100% (for example, sequence length = 4). From sequence length = 4 onwards, bilinguals had higher accuracy than monolinguals across the board (except for sequence length = 5, for which the accuracy of both groups was 85%). As the sequence length and consequently difficulty level of a block increased, the group differences became larger. The mean accuracy for monolinguals for the 6-, 7-, and 8- digit sequences was 52.8%, 31%, and 6%. For the same sequence lengths (in increasing order), the bilingual group’s overall accuracy was 62.7%, 49.4%, and 42.1%. A one-way analysis of variance showed that Accuracy was significantly affected by Group, F(1, 1731) = 23.67, *p* < 0.01 and Sequence Length, F(7, 1731) = 121.94, *p* < 0.01, and by these two factors’ interaction, F(6, 1731) = 4.15, *p* < 0.01. In a series of post hoc comparisons (with the Bonferroni correction) accuracy for list lengths 5 and 6 was significantly different from all of the other sequence lengths, while no significant differences were found in overall accuracy for sequence lengths 2, 3, and 4, and accuracy for sequence lengths 7 and 8 were significantly different from all other list lengths except for each other. A series of one-way ANOVAs performed separately for each list length showed significant effects of Group on Accuracy at sequence length 7, F(1, 157) = 5.62, *p* = 0.019 and sequence length 8, F(1, 51) = 6.75, *p* = 0.012. The results for Accuracy are very similar with those we obtained for the algorithm-based scores, so in the interest of space we will not be discussing the latter here, but only in the following section focusing on the correlations between variables.

**Figure 7:**
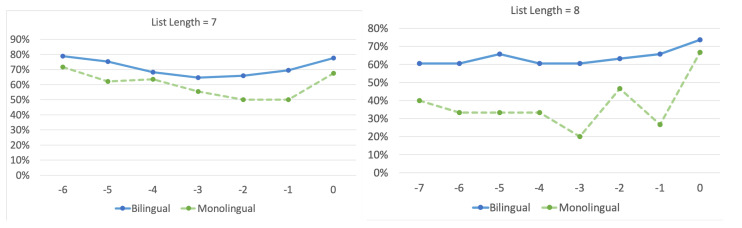
Digit span task: mean accuracy at each serial position for 7-digit (**right**) and 8-digit sequences (**left**). The terminal item is labeled with a 0, and each item preceding it is labeled in terms of its distance from the terminal item (e.g., −1 for the penultimate item, −2 for the antepenultimate item, etc.).

Figure 7 takes a closer look at the 7- and 8-digit sequences by showing the two groups’ mean accuracy at each serial position. A small number of the participants’ responses had to be excluded in order to create these plots in cases where the length of the response differed from the length of the input (for example, shorter responses such as “46,382” when the input had been “95,164,832”).

For both sequence lengths, we observe small primacy as well as recency effects, as for both of the groups the accuracy for initial and final items tended to be higher than that of items from the middle of the sequence, except for the initial items in the 8-digit sequence for the bilinguals. For the sequences containing 7 digits, bilinguals display higher accuracy than monolinguals at all serial positions, a difference that is smaller for the initial items but gradually becomes larger for each position that follows them through the preterminal position. In final position, probably because of a recency effect, the two groups perform more similarly than in the penultimate position.

Moving on to the sequences comprising 8 digits, we observe a similar pattern to that described above for 7-digit sequences. Bilinguals have higher accuracy than monolinguals at all serial positions and recency effects are noted for both groups, while only monolinguals display a slight primacy effect. Other patterns that can be observed include the overall lower accuracy for both groups (as might be expected due to the increased difficulty of having to recall the longer sequence), and the larger difference in group performance at all serial positions. Monolinguals show a spike in accuracy for the antepenultimate position, not noted with the 7-digit sequence length. This may have to do with individual factors, considering that only about 10% of the monolingual participants were able to reach this sequence length.

#### 2.6.3. Correlations between Phonetic and Phonological Learning and Auditory Sensory Memory

Lastly, we consider the potential link between phonetic and phonological learning performance and auditory sensory memory. Correlation analyses were performed using the following variables:

- **Phonetic and phonological learning:** Accent Score (both Overall, collapsing performance on the Training and Testing blocks, and separately for each of these two blocks)
- **Auditory sensory memory:** Maximum Sequence Length reached, Overall Digit Accuracy obtained in the memory task, and Algorithm-based Score, that is, the digit recall score obtained by taking into account permutation errors, with bigger penalties for items displaced at longer distances.

Table 1 shows the Pearson correlations that were significant in a two-tailed analysis when monolinguals and bilinguals (n = 60) were considered together, while Table 2 and Table 3 show the Pearson correlations that were significant in a one-tailed analysis when monolinguals and bilinguals were considered separately (n = 30 for each group).

**Figure 8:**
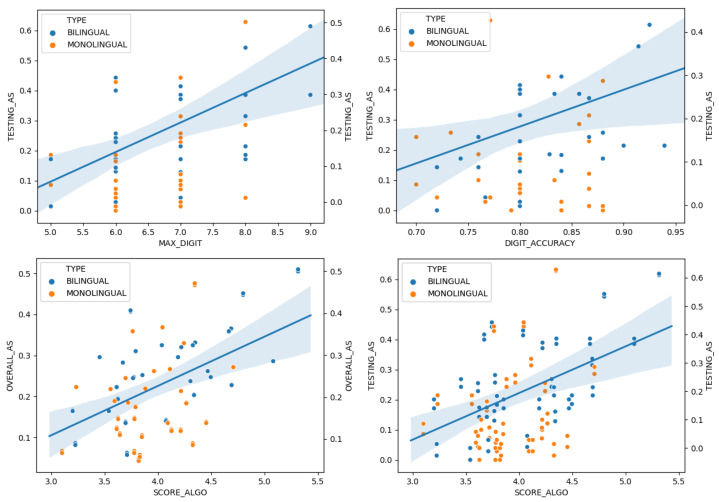
Regression plots for pairs of variables reflecting phonetic and phonological learning (Testing AS, Overall AS) and variables associated with auditory sensory memory (Max Digit, Digit Accuracy, and Score Algo). AS = accent score. Max Digit = maximum sequence length reached. Digit Accuracy = overall accuracy obtained in the digit span task, Score Algo = the corrected algorithm-based score obtained by taking into account permutation errors, with bigger penalties for items displaced at longer distances.

To summarize the above, several significant correlations were found between variables associated with phonetic and phonological learning and auditory sensory memory (and more generally serial memory, given the difficulty of excluding the effects of working memory). Specifically, the accent scores obtained by participants in both the training (imitation) condition and in testing, as well as (in some cases) the compounded overall scores, correlated with the maximum digit span, overall digit accuracy, and corrected algorithm scores obtained by the same participants. These correlations, however, were stronger and more numerous in the bilingual group. When considered separately, only three positive correlations were significant for the monolingual group, all of which were weak correlations. For comparison, 9 correlations were significant based on the data from bilingual speakers, of which 7 were strong correlations and 2 were of moderate strength. Figure 8 provides visual representations for some of these correlations.

## 3. Discussion

Our study supports the hypotheses we formulated, replicating earlier results (Hypothesis 1, [2,27,28,29] and Hypothesis 2, [46]) and also reporting a novel finding (Hypothesis 3). Specifically, bilinguals obtained higher performance scores on the novel accent learning task for all four features tested (Hypothesis 1). This was most apparent in the testing phase, but bilinguals also outperformed monolinguals in the training (imitation) block for three of the four features. More generally, bilinguals also obtained higher scores than monolinguals on sentences containing 1, 2, or 4 different features, with monolinguals showing a return to baseline in the more complex case of sentences requiring all four features to be expressed, reflecting an inability to manifest the newly learned patterns even though they were able to produce them in isolation. Both bilinguals and monolinguals performed better on the novel intonation pattern in tag questions than on the other three patterns, possibly due to the fact that it was a more global (suprasegmental) phenomenon of longer duration and higher salience compared to the other features.

Hypothesis 2 was supported by the finding that there is a bilingual advantage in auditory sensory memory (manifested as better performance on the items that preceded the suffix, see Figure 7), which became more pronounced as the task’s complexity (i.e., the length of the sequence to be recalled) increased. This suggests bilinguals have a longer auditory sensory memory span than monolinguals. This assumption is also supported by the percentage of participants in each group who reached the longest digit sequences (that is, 8 and 9 digits), and the two groups’ performance in terms of accuracy both when we considered the sequences as a whole and when we broke them down by serial position. Notably, the increase from 7- to 8-digit sequences caused a substantial drop in accuracy in the monolingual group (from 31% to 6%), while the decrease in accuracy was more gradual in bilinguals (from 49.4% to 42.1%). Additionally, the higher accuracy exhibited by bilinguals with the items positioned at the start of longer sequences suggests potential enhancement of their working memory as well, in line with earlier behavioral [45,52,53,54], and electrophysiological findings [55], but in contrast with studies which found no differences in working memory between bilinguals and monolinguals [56,57]. Lastly, Hypothesis 3 was also supported, as significant positive correlations were found between variables reflecting phonetic and phonological learning and variables associated with auditory sensory memory. We found this relationship to be much stronger in bilinguals compared to monolinguals.

One of the immediately arising questions in light of our results is whether the fact that the link between phonetic and phonological learning and auditory sensory memory was much stronger in bilinguals supports the idea that auditory sensory memory plays a crucial part in this type of learning. While we believe this to be the case, based on arguments we discuss in what follows, we would like to clarify that our study has not investigated the existence of a causal relationship between the two, but simply established that a relationship exists. The possibility remains that bilingual experience leads to the independent enhancement of both phonetic and phonological learning on the one hand and auditory sensory memory on the other hand, without the former being supported by the latter. Future studies are needed to elucidate this question.

In support of the involvement of auditory sensory memory in phonetic and phonological learning, Calabrese [18] discusses a mechanism involving two distinct modes of speech perception, the phonemic and the phonetic mode [58]. Listeners are posited to engage in the top-down, “phonemic” mode of perception when they perceive stimuli containing native-language phonological categories. This mode enables rapid unfolding of speech perception because it is able to ignore non-contrastive aspects of perceptual representations. But if perception were exclusively phonemic, that would mean that listeners are unable to to perceive allophonic variation, which would make languages unlearnable. It is the “phonetic” (or bottom-up) perception that enables access to allophonic details. “Phonetically-relevant perception” is thus crucial in order to learn allophonic variation and access sound contrasts in both native and non-native languages, as well as for acquiring foreign sounds. To achieve this, the perceptual system is assumed to contain a memory component for preserving acoustically accurate representations of the received signal making it possible for novel representations to be stored in order to (eventually) construct a new phonological system. While Calabrese uses the term echoic memory for this specific type of memory, it has more recently been referred to as auditory sensory memory [36]. A part of the bottom-up perceptual component, auditory sensory memory is posited to play a part in language learning (and more specifically in phonetic and phonological learning). From this perspective, the concept of phonological “deafening” for adults (to non-native sounds) does not describe an inability to hear or access the acoustic signal. Instead, it refers to their inability to translate the new cue pattern characterizing the non-native sound into a permissible phonological representation. Crucially, auditory sensory memory makes it possible for these novel acoustic patterns to be heard and preserved. Following sufficient articulatory training, acoustic patterns captured by auditory sensory memory can eventually be adapted into admissible phonological representations, at which point a learner has become able to acquire the non-native sound.

Other studies have acknowledged the role played by sensorimotor systems in language learning. Earlier findings [59,60] point to the existence of a specific left lateralized auditory mirror neuron system engaged in auditorily triggered speech imitation which [19] found be more active in “poor” speech imitators.Simmonds et al. (2011) also discuss how learning to speak a second language also has effects on auditory and somatosensory feedback systems, and emphasize the motor and sensory complexities involved in learning to speak a second language as an adult [21]. Their suggestion is that adult second language learners might benefit from a mute period of intense auditory exposure to a second language before attempting to produce the sounds. This mute period could prove to be “beneficial in enabling the learner to hear (and thus produce) subtly different phonetic features, new phoneme distinctions and unfamiliar sequences of stress patterns”. Future neurolinguistic findings may shed more light in this respect, also taking into account the involvement of the insula region, which was identified as a key component of accent processing, possibly playing a role in sensory-perceptual processing [61], and supporting conscious awareness and regulation of accent features [62]. This may help in understanding the observed differences between monolinguals and bilinguals in phonetic and phonological learning because these differences may partially also be due to the two groups’ recruiting different cognitive resources to achieve learning, with more conscious and effortful processing in the case of monolinguals.

Other than the relatively reduced number of participants, our study is subject to the methodological limitations we have pointed out in the introduction, such as not being able to obtain homogenous groups of bilingual speakers with respect to their experience with each of the languages they speak [6]. Given the lack of a unitary definition of “bilingual”, two people with very similar linguistic backgrounds and abilities might readily place themselves in different groups [63]. Among many possible scenarios, speakers might not feel confident enough to report bilingual knowledge if the second language is mostly practiced passively (e.g., their parents speak it at home but they only speak it occasionally), if they do not have the same competencies as their native-speaking relatives (e.g., a heritage speaker of Chinese or Arabic in the United States might not consider themselves bilingual because they cannot read or write in this language), or if the second language they speak is in some ways similar to another one, to the point where they feel they speak a somehow inferior version of that language (e.g., Haitian Creole speakers reporting they speak “broken French”). Other problems with self-reports include the fact that speakers may not accurately record the age of first exposure to a given language or how often and in what ways they were exposed to it (e.g., they may not be aware of extended trips abroad in their early childhood) and thus under-report their experience. While all of our bilingual participants reported (near-) native competence in both languages, high variability emerged in their performance on the short translation task administered at the beginning of the experiment (the analysis of which we have not yet completed). This inability to control for bilingual experience has been acknowledged as a major challenge in the study of bilingual cognition, thus future studies may benefit from the use of standardized language tests in order to evaluate a speaker’s proficiency. If such tests enabling finer-grained assessment of bilingual abilities are incorporated to experimental procedures, this may result in higher replicability, rendering more comparable the results of different studies [64]). Very recent work in neurolinguistics also supports this position as it indicates that proficiency (even more than age of acquisition)—is a critical factor differentiating the functional organization of bilingual language processing, a finding which has also been “underlined by structural neuroimaging investigations” [65].

Despite the mean group differences, in the current study we saw a number of monolinguals performing as well as the top bilinguals, for instance the top 10 performers on the novel accent learning task included 3 monolinguals, as did the top 10 participants with the highest digit span reached. In terms of overall accuracy on the digit span, 4 monolinguals were among the top 10 performers. This highlights another methodological complication: other than the use of multiple languages, several factors have been found to modulate the development of cognitive functioning, including socio-economic status [66], physical activity [67], circadian rhythm and sleep [68], dietary intake [69], and musical expertise [70]. Language learning constitutes one out of several possible ways of engaging in cognitive training, and cognitive training itself is only one of the lifestyle factors also known to affect cognitive function [8]. Studying any one of these aspects in isolation might obscure other meaningful relationships or be subject to confounds preventing us from observing significant effects, and contributing to replication failure. According to [71], we may expect future work to uncover that distinct effects of language on cognitive operations arise from interdependent functions. In consequence, research studies exploring directly how multiple levels of processing interact with one another have the potential to offer a more far-reaching view of how exactly language shapes our mind.

## 4. Conclusions

Our study focused on the sensorimotor bases of language—and more specifically phonetic and phonological—learning in bilinguals. We replicated the bilingual advantage previously observed in phonetic and phonological learning [2,27,28,29] and auditory sensory memory [46], though the differential roles of working memory and auditory sensory memory have yet to be determined more precisely. Our study also showed a significant correlation between phonetic and phonological learning and auditory sensory memory, which was stronger in bilinguals in comparison to monolinguals. Whereas higher-level cognitive functions are likely to be at play in the execution of complex tasks such as phonetic and phonological learning, it is important not to underestimate the role played by lower-level, sensorimotor functions as well, so a full picture of the mechanism supporting this type of learning may be obtained. These findings thus raise questions about the role of sensorimotor mechanisms in language learning and suggest that incorporating a sensorimotor perspective in future studies on bilingual cognition may be a fruitful research direction.

## Figures and Tables

**Table 1:** Significant correlations between variables associated with phonetic and phonological learning (arranged vertically) and variables associated with auditory sensory memory (arranged horizontally) when all participants were considered together. Gray shading indicates the strength of the correlation (light gray = weak correlation, medium gray = moderate correlation, dark gray = strong correlation); n.s. = not significant.

	Max Sequence Length	Overall Accuracy	Algorithm-Based Score
**Accent Score** **(Overall)**	r(58) = 0.497 *p* < 0.001	n.s.	r(58) = 0.504 *p* < 0.001
**Accent Score** **(Testing)**	r(58) = 0.479 *p* < 0.001	r(58) = 0.297 *p* < 0.05	r(58) = 0.469 *p* < 0.001
**Accent Score** **(Training)**	r(58) = 0.445 *p* < 0.001	r(58) = 0.312 *p* < 0.05	r(58) = 0.459 *p* < 0.001

**Table 2:** Significant correlations between variables associated with phonetic and phonological learning (arranged vertically) and variables associated with auditory sensory memory (arranged horizontally) for the MONOLINGUAL group. Gray shading indicates the strength of the correlation (light gray = weak correlation, medium gray = moderate correlation, dark gray = strong correlation); n.s. = not significant.

	Max Sequence Length	Overall Accuracy	Algorithm-Based Score
**Accent Score** **(Overall)**	r(28) = 0.379 *p* < 0.05	n.s.	n.s.
**Accent Score** **(Testing)**	r(28) = 0.370 *p* < 0.05	n.s.	n.s.
**Accent Score** **(Training)**	r(28) = 0.336 *p* < 0.05	n.s.	n.s.

**Table 3:** Significant correlations between variables associated with phonetic and phonological learning (arranged vertically) and variables associated with auditory sensory memory (arranged horizontally) for the BILINGUAL group. Gray shading indicates the strength of the correlation (light gray = weak correlation, medium gray = moderate correlation, dark gray = strong correlation); n.s. = not significant.

	Max Sequence Length	Overall Accuracy	Algorithm-Based Score
**Accent Score** **(Overall)**	r(28) = 0.572 *p* < 0.001	r(28) = 0.525 *p* = 0.001	r(58) = 0.617 *p* < 0.001
**Accent Score** **(Testing)**	r(28) = 0.539 *p* = 0.001	r(28) = 0.518 *p* < 0.05	r(28) = 0.581 *p* < 0.001
**Accent Score** **(Training)**	r(28) = 0.499 *p* < 0.05	r(28) = 0.451 *p* < 0.05	r(28) = 0.527 *p* < 0.001

## Data Availability

Not applicable.

## Appendix A

**VARIABLES MEASURING PHONETIC AND PHONOLOGICAL LEARNING**

**Accent Score (Overall)** The absence or presence of the four novel features associated with the Model Speech (in the appropriate environment) was scored with a 0 or 1, respectively. This was achieved for all three blocks completed by the participants (Baseline, Training, and Testing). Subsequently, an overall score was obtained for each participant, indicating the percentage of novel features produced (out of their total utterances). Note that the features were not expected to appear in the Baseline block which reflected participants’ natural accents.
**Accent Score (Training)** The absence or presence of the four novel features associated with the Model Speech (in the appropriate environment) was scored with a 0 or 1, respectively. This was achieved separately for the Training block, which consisted of participants’ imitation of sentences uttered in the Model Speech accent immediately after hearing each one of them. Subsequently, an overall score was obtained for each participant, indicating the percentage of novel features produced during Training (out of their total utterances).
**Accent Score (Testing)** The absence or presence of the four novel features associated with the Model Speech (in the appropriate environment) was scored with a 0 or 1, respectively. This was achieved separately for the Testing block, which consisted of participants’ re-reading of the sentences presented during the Baseline block, being prompted to now utter them in the Model Speech accent they had received training on, to the best of their ability. Subsequently, an overall score was obtained for each participant, indicating the percentage of novel features produced during Testing (out of their total utterances).

**VARIABLES MEASURING AUDITORY SENSORY MEMORY**

**Max Sequence Length** This variable measures the longest digit sequence reached by a participant. The first digit sequence presented contained two digits only (and also served as a practice block) following which, if a participant correctly recalled at least 3 out of a total of 5 trials per block, the next digit sequence would be presented (one digit longer than the one that had just been completed). The task was adaptive therefore this part of the experiment would end whenever a participant failed to successfully recall at least 3 trials for a given sequence.
**Overall Digit Accuracy** A participant’s overall accuracy in the digit span task. Since each sequence length included 5 trials, errors were possible even when participants were able to advance successfully to the next sequence length. Participants from both groups started making errors from sequence length = 4 and higher.
**Algorithm-based Accuracy Score** A ’corrected’ score that took into account the similarity between a digit sequence input and a participant’s response. Thus, a response string that was very similar to the input (for instance by the transposition of 2 digits) received a higher score than a response in none of the digits matched the input (the Overall Digit Accuracy would have assigned both such sequences an identical score of 0). The algorithm searched for insertions or deletions by aligning a participant response string and the input string presented and counting the number of digits in each string to see if there was a discrepancy. If the number of digits in the response string was equal to the number of digits in the input string, then the answer was included in the analysis, but if the number of digits was not equal between the response and input strings, the answer was excluded. The algorithm evaluated the responses that were included digit-by-digit, employing a graded scoring method that assigned weighted scores based on transpositional distance. The goal of this graded scoring system was to award a higher score to transposed response digits that were closer to their original position in the participant response.
